# The genome sequence of fat-hen, *Chenopodium album* L.

**DOI:** 10.12688/wellcomeopenres.23015.1

**Published:** 2024-09-04

**Authors:** Sahr Mian, Maarten J. M. Christenhusz, Royal Botanic Gardens Kew Genome Acquisition Lab, Plant Genome Sizing collective, Darwin Tree of Life Barcoding collective, Wellcome Sanger Institute Tree of Life Management, Samples and Laboratory team, Wellcome Sanger Institute Scientific Operations: Sequencing Operations, Wellcome Sanger Institute Tree of Life Core Informatics team, Tree of Life Core Informatics collective, Darwin Tree of Life Consortium

**Affiliations:** 1 Royal Botanic Gardens Kew, Richmond, England, UK; 2 Curtin University, Perth, Western Australia, Australia

**Keywords:** Chenopodium album, fat-hen, genome sequence, chromosomal, Caryophyllales

## Abstract

We present a genome assembly from an individual *Chenopodium album* (fat-hen; Streptophyta; Magnoliopsida; Caryophyllales; Chenopodiaceae). The genome sequence has a total length of 1,593.80 megabases. Most of the assembly is scaffolded into 27 chromosomal pseudomolecules suggesting the individual is an allohexaploid (2 *n* = 6 *x* = 56). The mitochondrial and plastid genome assemblies have lengths of 312.95 kilobases and 152.06 kilobases, respectively. Gene annotation of this assembly on Ensembl identified 50,077 protein-coding genes.

## Species taxonomy

Eukaryota; Viridiplantae; Streptophyta; Streptophytina; Embryophyta; Tracheophyta; Euphyllophyta; Spermatophyta; Magnoliopsida; Mesangiospermae; eudicotyledons; Gunneridae; Pentapetalae; Caryophyllales; Chenopodiaceae; Chenopodioideae; Atripliceae; *Chenopodium*; *Chenopodium album* L. (NCBI:txid3559).

## Background

*Chenopodium album* L., is an annual herb of disturbed ground, waste places, gardens, roadsides and fields. It is called ‘white goosefoot’ due to the shape of the leaves, or ‘fat-hen’ because it was commonly grown in Britain and elsewhere in Europe as a food for chickens. The plant has long been in use as a food plant, often prepared in a similar fashion to the closely related spinach ( *Spinacia oleracea* L.) as it tastes much the same. The area of origin is uncertain, but it seems to be native across Eurasia and possibly also in eastern parts of North America ( POWO, 2024). It is also common across Asia, including in the Indian subcontinent where it is an important plant in local cuisine and known as ‘bathua’ (see Singh *et al.*, 2023). It is widely naturalised in northern Europe, southern Africa, Australasia and the Americas ( POWO, 2024).

Fat-hen is an annual herb with alternate leaves that vary in shape and size but are toothed and deltoid in shape and become entire and lanceolate in the inflorescence. The entire plant is coated in a white, mealy wax, especially prominent on the lower leaf surface and the stems, giving the plant a grey-green appearance. Flowers are bisexual or female – the two types mixed in the same inflorescence – with five green tepals and five stamens when present ( Stace *et al.*, 2019).

Both seeds and leaves have been consumed by humans since ancient times in Europe and Asia, with remains having been found in Iron Age, Viking and Roman settlements across the continents ( Behre, 2008; Renfrew, 1973). Seeds have also been found in the stomachs of bog bodies in Denmark ( Miles, 1978; Robinson, 1994), and are useful in carbon dating of archaeological sites, because they are resilient in the soil ( Alanko *et al.*, 2015).

Even though it is a useful plant as both seeds and leaves are edible, it is also a problematic weed in agriculture, capable of causing major crop losses due to competition and allelopathic interactions ( Bajwa *et al.*, 2019; Reinhardt *et al.*, 1994). It is also a host to the beet leafhopper ( *Circulifer tenellus*) that may transmit curly-top virus ( *Curtovirus*) to beet crops ( Bennett, 1971; Severin & Henderson, 1928). In addition, pollen of *Chenopodium album* may contribute to allergies, particularly in dry regions and in urban areas of high pollution ( Amjad & Shafighi, 2012; Nouri *et al.*, 2012).

Numerous microspecies (or subspecies/varieties) have been recognised, but these are difficult to differentiate and are not generally accepted. For this study, we sampled a typical plant from an allotment in southwest London, where it grows as a common weed among cultivated crops.

Although both diploid (2 *n* = 2 *x* = 18) and hexaploid (2 *n* = 6 *x* = 54) cytotypes of C. album have been reported in the UK based on a basic chromosome number of *x* = 9, but hexaploids are by far the most common (e.g. Rahiminejad & Gornall, 2004). Recent cytogenetic and genomic analyses have uncovered the complex genomic origin of the hexaploid *C. album*, confirming that it is an allopolyploid ( Krak *et al.*, 2016; Mandák *et al.*, 2012; Mandák *et al.*, 2018).

The high-quality genome presented here will be useful for understanding the taxonomy of the species. It may also help in developing the species as a climate-resilient food crop, while potentially helping to understand allelopathy and allergens in this species.

## Genome sequence report

**Figure 1.**
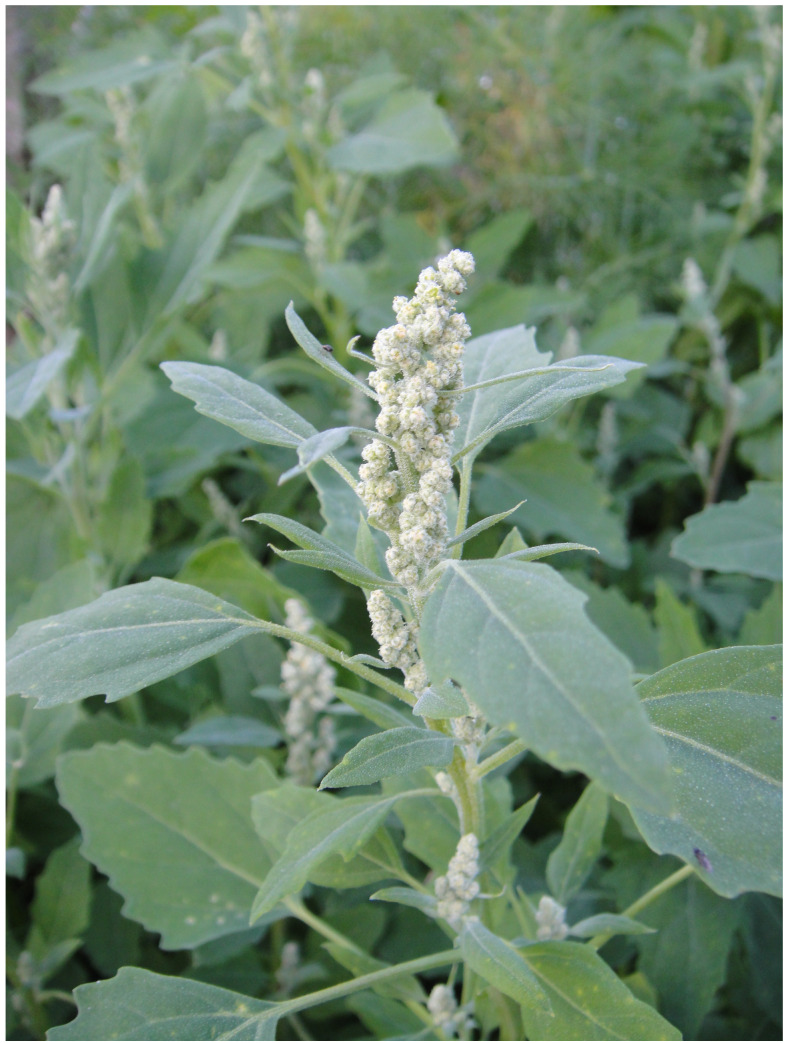
Photograph of the *Chenopodium album* (dcCheAlbu1) specimen used for genome sequencing.

The genome of *Chenopodium album* ( Figure 1) was sequenced using Pacific Biosciences single-molecule HiFi long reads, generating a total of 21.85 Gb (gigabases) from 1.84 million reads, providing approximately 53-fold coverage. Using flow cytometry, the genome size (1C-value) was estimated to be 2.09 pg, equivalent to 2,040 Mb. Primary assembly contigs were scaffolded with chromosome conformation Hi-C data, which produced 233.39 Gb from 1,545.60 million reads, yielding an approximate coverage of 146-fold. Specimen and sequencing information is summarised in Table 1.

**Table 1.** Specimen and sequencing data for *Chenopodium album*.

Project information			
**Study title**	Chenopodium album (white goosefoot)		
**Umbrella BioProject**	PRJEB58230		
**Species**	*Chenopodium album*		
**BioSample**	SAMEA9335283		
**NCBI taxonomy ID**	3559		
Specimen information			
**Technology**	**ToLID**	**BioSample** **accession**	**Organism** **part**
**PacBio long read sequencing**	dcCheAlbu1	SAMEA9335451	leaf
**Hi-C sequencing**	dcCheAlbu1	SAMEA9335451	leaf
**RNA sequencing**	dcCheAlbu1	SAMEA9335459	leaf
**Sequencing information**			
**Platform**	**Run accession**	**Read count**	**Base count** **(Gb)**
**Hi-C Illumina NovaSeq 6000**	ERR10684069	1.55e+09	233.39
**PacBio Sequel IIe**	ERR10677844	1.39e+06	12.52
**PacBio Sequel IIe**	ERR10684181	1.53e+06	13.96
**PacBio Sequel IIe**	ERR10677843	1.84e+06	21.85
**RNA Illumina NovaSeq 6000**	ERR10684070	4.15e+07	6.27

**Figure 2.**
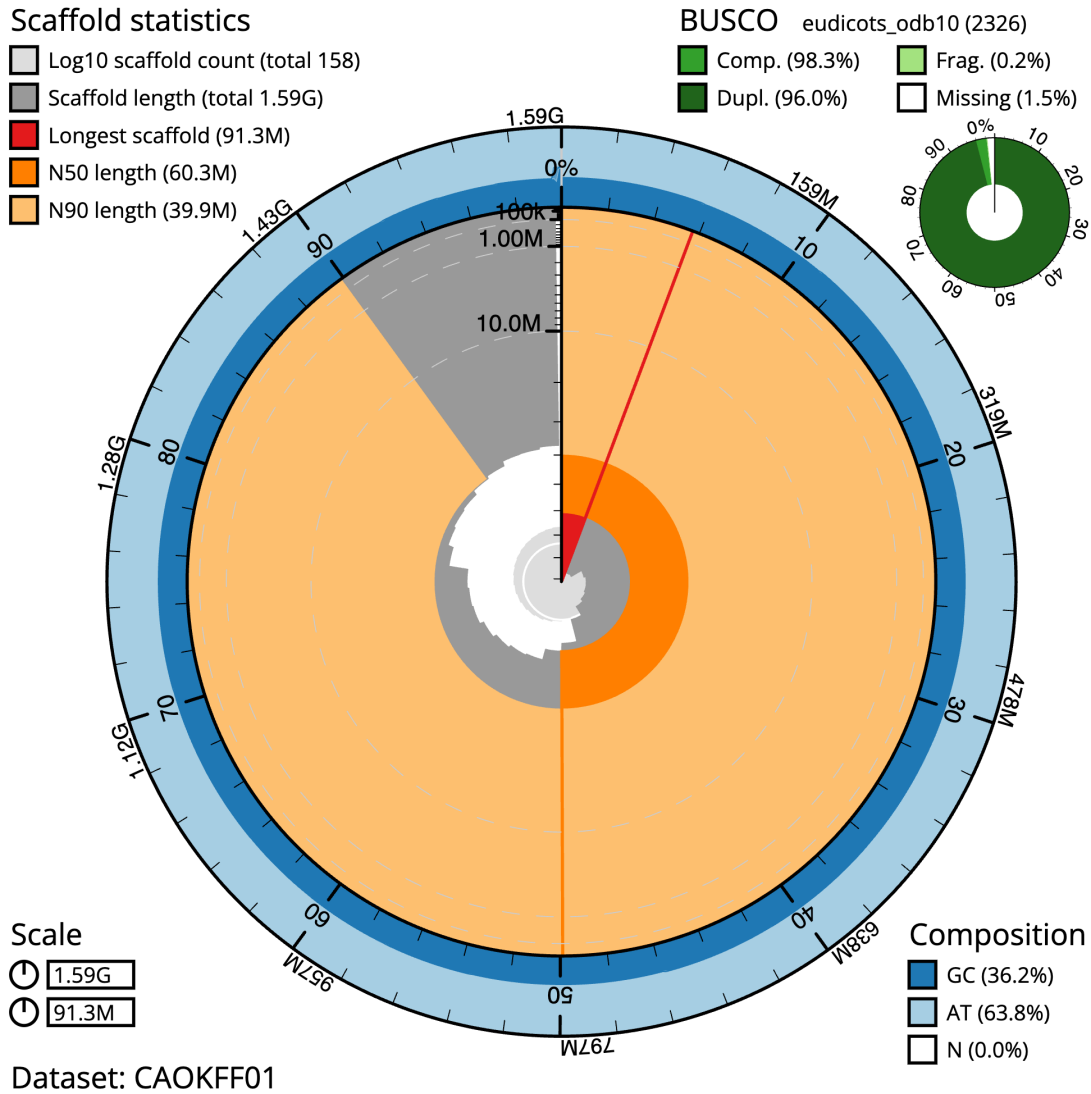
Genome assembly of *Chenopodium album*, dcCheAlbu1.1: metrics. The BlobToolKit snail plot shows N50 metrics and BUSCO gene completeness. The main plot is divided into 1,000 size-ordered bins around the circumference with each bin representing 0.1% of the 1,594,308,297 bp assembly. The distribution of scaffold lengths is shown in dark grey with the plot radius scaled to the longest scaffold present in the assembly (91,301,842 bp, shown in red). Orange and pale-orange arcs show the N50 and N90 scaffold lengths (60,299,674 and 39,868,957 bp), respectively. The pale grey spiral shows the cumulative scaffold count on a log scale with white scale lines showing successive orders of magnitude. The blue and pale-blue area around the outside of the plot shows the distribution of GC, AT and N percentages in the same bins as the inner plot. A summary of complete, fragmented, duplicated and missing BUSCO genes in the eudicots_odb10 set is shown in the top right. An interactive version of this figure is available at https://blobtoolkit.genomehubs.org/view/CAOKFF01/dataset/CAOKFF01/snail.

**Figure 3.**
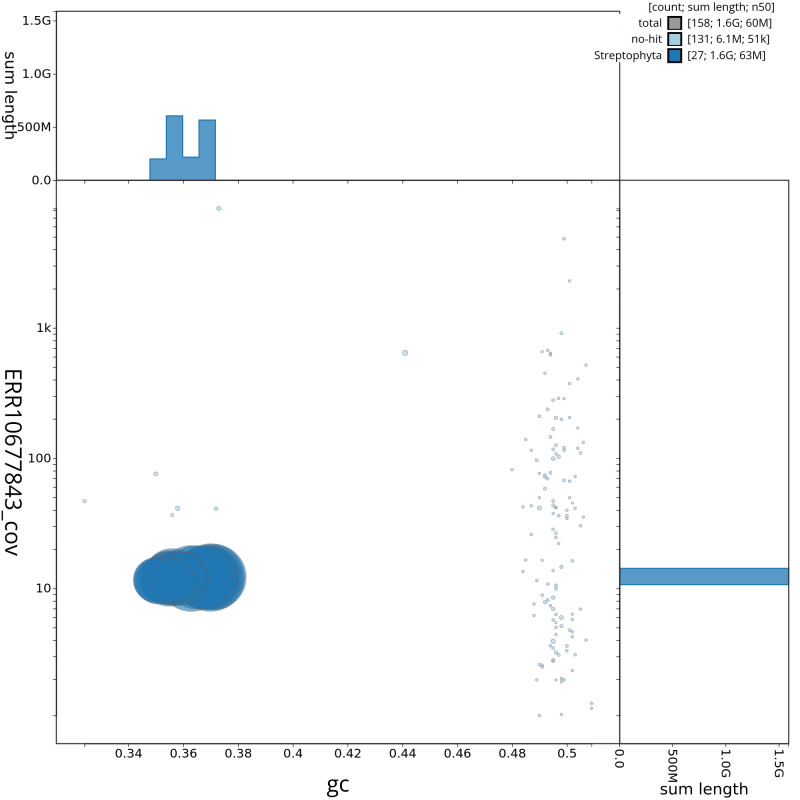
Genome assembly of *Chenopodium album*, dcCheAlbu1.1: Distribution plot of base coverage in ERR10677843 against position for sequences along each of the 27 assembled chromosomes in the assembly CAOKFF01. Windows of 100kb are coloured by phylum. The assembly has been filtered to exclude sequences with length < 2,550,000. An interactive version of this figure is available here.

**Figure 4.**
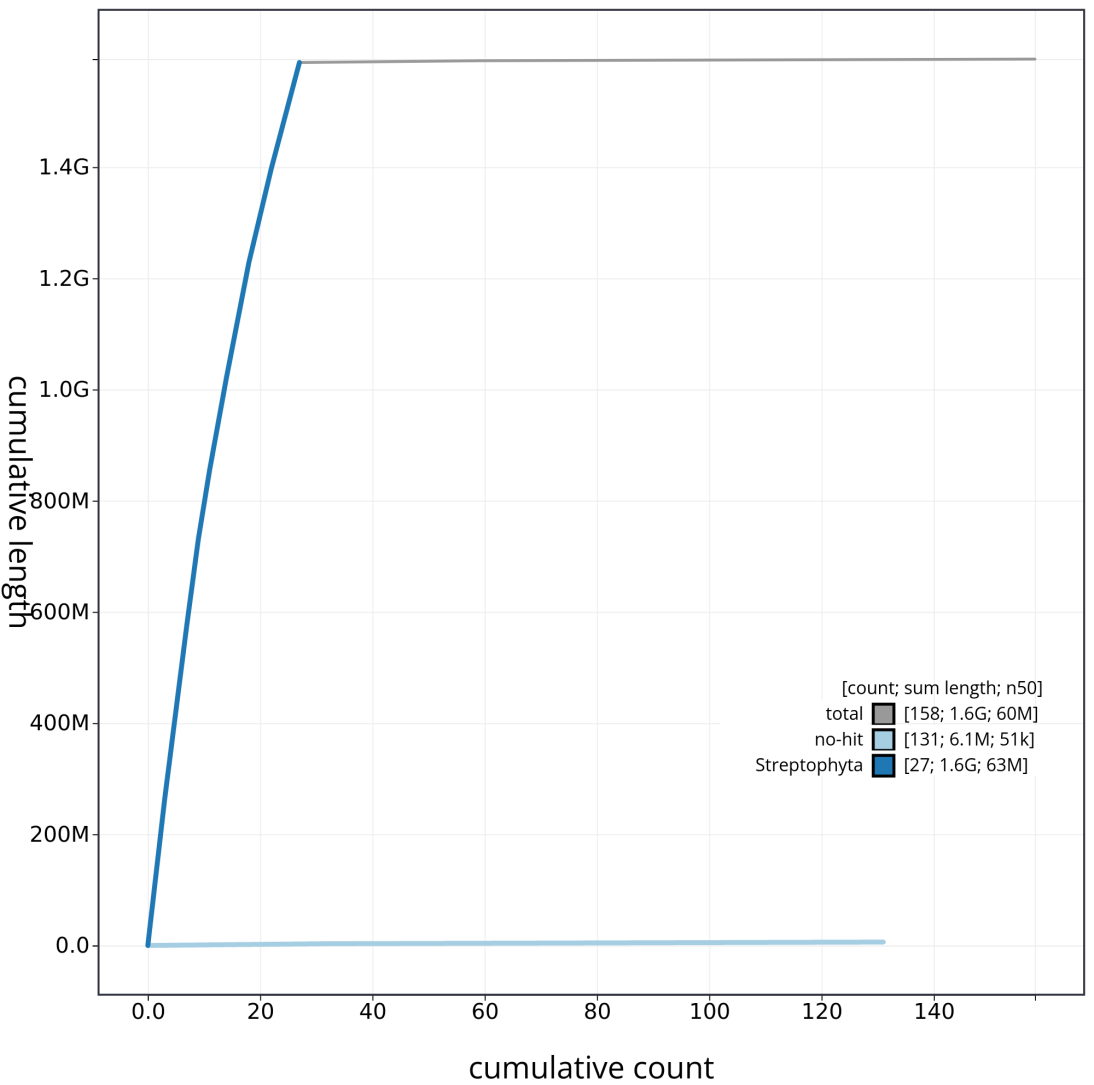
Genome assembly of *Chenopodium album*, dcCheAlbu1.1: BlobToolKit cumulative sequence plot. The grey line shows cumulative length for all scaffolds. Coloured lines show cumulative lengths of scaffolds assigned to each phylum using the buscogenes taxrule. An interactive version of this figure is available at https://blobtoolkit.genomehubs.org/view/CAOKFF01/dataset/CAOKFF01/cumulative.

**Figure 5.**
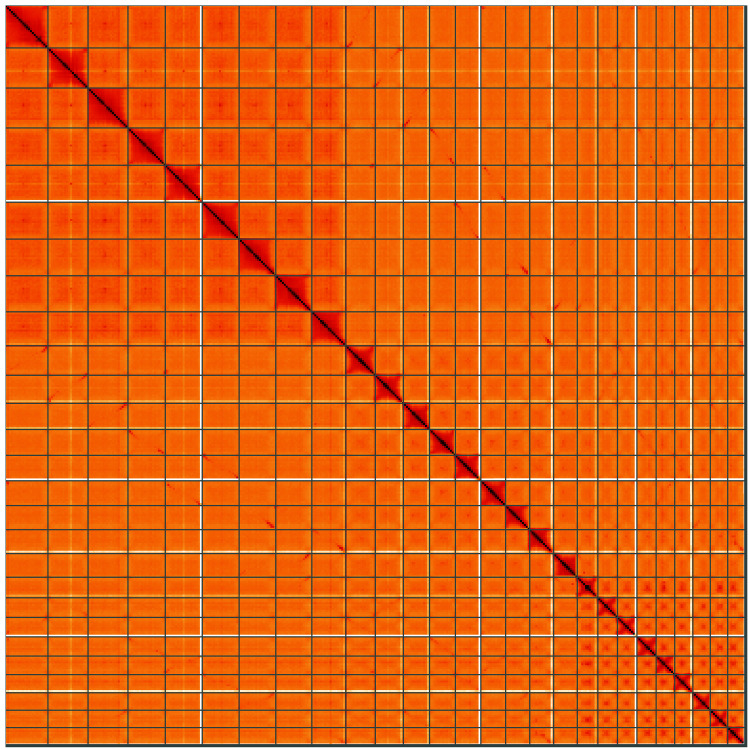
Genome assembly of *Chenopodium album*, dcCheAlbu1.1: Hi-C contact map of the dcCheAlbu1.1 assembly, visualised using HiGlass. Chromosomes are shown in order of size from left to right and top to bottom. An interactive version of this figure may be viewed at https://genome-note-higlass.tol.sanger.ac.uk/l/?d=QH1Co6i1RL6k53dsnNEL-A.

Manual assembly curation corrected three missing joins or mis-joins. The final assembly has a total length of 1,593.80 Mb in 156 sequence scaffolds with a scaffold N50 of 60.3 Mb ( Table 2). The snail plot in Figure 2 provides a summary of the assembly statistics, while Figure 3 shows the base coverage against position in each chromosome. The cumulative assembly plot in Figure 4 shows curves for subsets of scaffolds assigned to different phyla. Most (99.61%) of the assembly sequence was assigned to 27 chromosomal-level scaffolds. Chromosome-scale scaffolds confirmed by the Hi-C data are named in order of size ( Figure 5; Table 3). Given that the genome sequence assembles into 27 unique chromosome-level scaffolds, the data suggest the individual sequenced is an allohexaploid (2 *n* = 6 *x* = 54). The mitochondrial and plastid genomes were also assembled and can be found as contigs within the multifasta file of the genome submission.

**Table 2.** Genome assembly data for *Chenopodium album*, dcCheAlbu1.1.

Genome assembly		
Assembly name	dcCheAlbu1.1	
Assembly accession	GCA_948465745.1	
*Accession of alternate* *haplotype*	*GCA_948466375.1*	
Span (Mb)	1,593.80	
Number of contigs	225	
Contig N50 length (Mb)	31.5	
Number of scaffolds	156	
Scaffold N50 length (Mb)	60.3	
Longest scaffold (Mb)	91.3	
Assembly metrics *		*Benchmark*
Consensus quality (QV)	67.6	*≥ 50*
*k*-mer completeness	100.0%	*≥ 95%*
BUSCO **	C:98.3%[S:2.3%,D:96.0%], F:0.2%,M:1.5%,n:2,326	*C ≥ 95%*
Percentage of assembly mapped to chromosomes	99.61%	*≥ 95%*
Organelles	Mitochondrial genome: 312.95 kb; plastid genome: 152.06 kb	*complete single alleles*
Genome annotation at Ensembl		
Number of protein-coding genes	50,077	
Number of non-coding genes	18,072	
Number of gene transcripts	84,147	

* Assembly metric benchmarks are adapted from column VGP-2020 of “Table 1: Proposed standards and metrics for defining genome assembly quality” from Rhie *et al.* (2021). ** BUSCO scores based on the eudicots_odb10 BUSCO set using version 5.4.3. C = complete [S = single copy, D = duplicated], F = fragmented, M = missing, n = number of orthologues in comparison. A full set of BUSCO scores is available at https://blobtoolkit.genomehubs.org/view/CAOKFF01/dataset/CAOKFF01/busco.

**Table 3.** Chromosomal pseudomolecules in the genome assembly of *Chenopodium album*, dcCheAlbu1.

INSDC accession	Name	Length (Mb)	GC%
OX419208.1	1	91.3	37.0
OX419209.1	2	86.14	36.5
OX419210.1	3	85.74	37.0
OX419211.1	4	80.21	37.0
OX419212.1	5	79.37	36.5
OX419213.1	6	79.26	37.0
OX419214.1	7	78.66	37.0
OX419215.1	8	77.47	37.0
OX419216.1	9	72.95	36.5
OX419217.1	10	63.43	35.5
OX419218.1	11	60.3	35.5
OX419219.1	12	56.1	36.0
OX419220.1	13	55.6	35.5
OX419221.1	14	54.62	35.5
OX419222.1	15	53.28	35.5
OX419223.1	16	51.76	35.5
OX419224.1	17	51.39	36.0
OX419225.1	18	50.82	36.0
OX419226.1	19	44.1	36.0
OX419227.1	20	42.23	35.5
OX419228.1	21	41.69	35.0
OX419229.1	22	41.29	35.5
OX419230.1	23	39.87	35.0
OX419231.1	24	39.0	35.0
OX419232.1	25	37.47	35.5
OX419233.1	26	37.36	35.0
OX419234.1	27	36.75	35.5
OX419235.1	MT	0.31	44.0
OX419236.1	Pltd	0.15	37.5

The estimated Quality Value (QV) of the final assembly is 67.6 with *k*-mer completeness of 100.0%, and the assembly has a BUSCO v5.4.3 completeness of 98.3% (single = 2.3%, duplicated = 96.0%), using the eudicots_odb10 reference set ( *n* = 2,326).

Metadata for specimens, BOLD barcode results, spectra estimates, sequencing runs, contaminants and pre-curation assembly statistics are given at https://links.tol.sanger.ac.uk/species/3559.

## Genome annotation report

The *Chenopodium album* genome assembly (GCA_948465745.1) was annotated at the European Bioinformatics Institute (EBI) on Ensembl Rapid Release. The resulting annotation includes 84,147 transcribed mRNAs from 50,077 protein-coding and 18,072 non-coding genes ( Table 2; https://rapid.ensembl.org/Chenopodium_album_GCA_948465745.1/Info/Index). The average transcript length is 3,378.52. There are 1.23 coding transcripts per gene and 4.58 exons per transcript.

## Methods

### Sample acquisition, DNA barcoding and genome size estimation

A specimen of *Chenopodium album* (specimen ID KDTOL10275, ToLID dcCheAlbu1) was collected from Kingston, Surrey, UK (latitude 51.43, longitude –0.30) on 2021-06-28. The specimen was collected by Sahr Mian and Maarten Christenhusz (both from the Royal Botanic Gardens Kew) and identified by Maarten Christenhusz and then frozen at –80 °C. The herbarium voucher associated with the sequenced plant is deposited in the herbarium of RBG Kew (K).

The initial species identification was verified by an additional DNA barcoding process according to the framework developed by Twyford *et al*. (2024). Part of the plant specimen was preserved in silica gel desiccant. A DNA extraction from the dried plant was amplified by PCR for standard barcode markers, with the amplicons sequenced and compared to public sequence databases including GenBank and the Barcode of Life Database (BOLD) as well as internal RBGE sequence databases. The barcode sequences for this specimen are openly available on BOLD ( Ratnasingham & Hebert, 2007). Following whole genome sequence generation, DNA barcodes were also used alongside the initial barcoding data for sample tracking through the genome production pipeline at the Wellcome Sanger Institute ( Twyford *et al.*, 2024). The standard operating procedures for the Darwin Tree of Life barcoding have been deposited on protocols.io ( Beasley *et al.*, 2023).

The genome size was estimated by flow cytometry at the Royal Botanic Gardens Kew using the fluorochrome propidium iodide and following the ‘one-step’ method as outlined in Pellicer *et al.* (2021). For this species, CyStain™ PI OxProtect Staining Buffer (cat. No. 05-5027; Sysmex UK Ltd.) was used for isolation of nuclei ( Loureiro *et al.*, 2007), and the internal calibration standard was *Pisum sativum* ‘Ctirad’ with an assumed 1C-value of 4,445 Mb ( Doležel *et al.*, 1998).

### Nucleic acid extraction

The workflow for high molecular weight (HMW) DNA extraction at the WSI Tree of Life Core Laboratory includes a sequence of core procedures: sample preparation; sample homogenisation, DNA extraction, fragmentation, and clean-up. Detailed protocols are available on protocols.io ( Denton *et al.*, 2023).

In sample preparation, the dcCheAlbu1 sample was weighed and dissected on dry ice ( Jay *et al.*, 2023). For sample homogenisation, leaf tissue was cryogenically disrupted using the Covaris cryoPREP ^®^ Automated Dry Pulverizer cryoPREP ^®^ Automated Dry Pulverizer ( Narváez-Gómez *et al.*, 2023). HMW DNA was extracted using the Automated Plant MagAttract v2 protocol ( Todorovic *et al.*, 2023). HMW DNA was sheared into an average fragment size of 12–20 kb in a Megaruptor 3 system ( Bates *et al.*, 2023). Sheared DNA was purified by solid-phase reversible immobilisation, using AMPure PB beads to eliminate shorter fragments and concentrate the DNA ( Strickland *et al.*, 2023). The concentration of the sheared and purified DNA was assessed using a Nanodrop spectrophotometer and Qubit Fluorometer and Qubit dsDNA High Sensitivity Assay kit. Fragment size distribution was evaluated by running the sample on the FemtoPulse system.

RNA was extracted from leaf tissue of dcCheAlbu1 in the Tree of Life Laboratory at the WSI using the RNA Extraction: Automated MagMax™ *mir*Vana protocol ( do Amaral *et al.*, 2023). The RNA concentration was assessed using a Nanodrop spectrophotometer and a Qubit Fluorometer using the Qubit RNA Broad-Range Assay kit. Analysis of the integrity of the RNA was done using the Agilent RNA 6000 Pico Kit and Eukaryotic Total RNA assay.

### Sequencing

Pacific Biosciences HiFi circular consensus DNA sequencing libraries were constructed according to the manufacturers’ instructions. Poly(A) RNA-Seq libraries were constructed using the NEB Ultra II RNA Library Prep kit. DNA and RNA sequencing was performed by the Scientific Operations core at the WSI on Pacific Biosciences Sequel IIe (HiFi) and Illumina NovaSeq 6000 (RNA-Seq) instruments. Hi-C data were also generated from leaf tissue of dcCheAlbu1 using the Arima-HiC v2 kit. The Hi-C sequencing was performed using paired-end sequencing with a read length of 150 bp on the Illumina NovaSeq 6000 instrument.

### Genome assembly, curation and evaluation

***Assembly***

The original assembly of HiFi reads was performed using the Hifiasm ( Cheng *et al.*, 2021) with the --primary option. Haplotypic duplications were identified and removed with purge_dups ( Guan *et al.*, 2020). Hi-C reads were further mapped with bwa-mem2 ( Vasimuddin *et al.*, 2019) to the primary contigs, which were further scaffolded using the provided Hi-C data ( Rao *et al.*, 2014) in YaHS ( Zhou *et al.*, 2023) using the --break option. Scaffolded assemblies were evaluated using Gfastats ( Formenti *et al.*, 2022), BUSCO ( Manni *et al.*, 2021) and MERQURY.FK ( Rhie *et al.*, 2020).

The organelle genomes were assembled using MitoHiFi ( Uliano-Silva *et al.*, 2023) and OATK ( Zhou, 2023).

***Curation***

The assembly was decontaminated using the Assembly Screen for Cobionts and Contaminants (ASCC) pipeline (article in preparation). Manual curation was primarily conducted using PretextView ( Harry, 2022), with additional insights provided by JBrowse2 ( Diesh *et al.*, 2023) and HiGlass ( Kerpedjiev *et al.*, 2018). Scaffolds were visually inspected and corrected as described by Howe *et al*. (2021). Any identified contamination, missed joins, and mis-joins were corrected, and duplicate sequences were tagged and removed. The process is documented at https://gitlab.com/wtsi-grit/rapid-curation (article in preparation).

***Evaluation of final assembly***

A Hi-C map for the final assembly was produced using bwa-mem2 ( Vasimuddin *et al.*, 2019) in the Cooler file format ( Abdennur & Mirny, 2020). To assess the assembly metrics, the *k*-mer completeness and QV consensus quality values were calculated in Merqury ( Rhie *et al.*, 2020). This work was done using the “sanger-tol/readmapping” ( Surana *et al.*, 2023a) and “sanger-tol/genomenote” ( Surana *et al.*, 2023b) pipelines. The genome readmapping pipelines were developed using the nf-core tooling ( Ewels *et al.*, 2020), use MultiQC ( Ewels *et al.*, 2016), and make extensive use of the Conda package manager, the Bioconda initiative ( Grüning *et al.*, 2018), the Biocontainers infrastructure ( da Veiga Leprevost *et al.*, 2017), and the Docker ( Merkel, 2014) and Singularity ( Kurtzer *et al.*, 2017) containerisation solutions. The genome was also analysed within the BlobToolKit environment ( Challis *et al.*, 2020) and BUSCO scores ( Manni *et al.*, 2021) were calculated.

Table 4 contains a list of relevant software tool versions and sources.

**Table 4.** Software tools: versions and sources.

Software tool	Version	Source
BlobToolKit	4.2.1	https://github.com/blobtoolkit/blobtoolkit
BUSCO	5.3.2	https://gitlab.com/ezlab/busco
bwa-mem2	2.2.1	https://github.com/bwa-mem2/bwa-mem2
Cooler	0.8.11	https://github.com/open2c/cooler
Gfastats	1.3.6	https://github.com/vgl-hub/gfastats
Hifiasm	0.16.1-r375	https://github.com/chhylp123/hifiasm
HiGlass	1.11.6	https://github.com/higlass/higlass
Merqury	MerquryFK	https://github.com/thegenemyers/MERQURY.FK
MitoHiFi	2	https://github.com/marcelauliano/MitoHiFi
OATK	0.1	https://github.com/c-zhou/oatk
PretextView	0.2	https://github.com/wtsi-hpag/PretextView
purge_dups	1.2.3	https://github.com/dfguan/purge_dups
sanger-tol/genomenote	v1.0	https://github.com/sanger-tol/genomenote
sanger-tol/readmapping	1.1.0	https://github.com/sanger-tol/readmapping/tree/1.1.0
YaHS	1.1a.2	https://github.com/c-zhou/yahs

### Genome annotation

The Ensembl Genebuild annotation system ( Aken *et al.*, 2016) was used to generate annotation for the *Chenopodium album* assembly (GCA_948465745.1) in Ensembl Rapid Release at the EBI. Annotation was created primarily through alignment of transcriptomic data to the genome, with gap filling via protein-to-genome alignments of a select set of proteins from UniProt ( UniProt Consortium, 2019).

### Wellcome Sanger Institute – Legal and Governance

The materials that have contributed to this genome note have been supplied by a Darwin Tree of Life Partner. The submission of materials by a Darwin Tree of Life Partner is subject to the **‘Darwin Tree of Life Project Sampling Code of Practice’**, which can be found in full on the Darwin Tree of Life website here. By agreeing with and signing up to the Sampling Code of Practice, the Darwin Tree of Life Partner agrees they will meet the legal and ethical requirements and standards set out within this document in respect of all samples acquired for, and supplied to, the Darwin Tree of Life Project.

Further, the Wellcome Sanger Institute employs a process whereby due diligence is carried out proportionate to the nature of the materials themselves, and the circumstances under which they have been/are to be collected and provided for use. The purpose of this is to address and mitigate any potential legal and/or ethical implications of receipt and use of the materials as part of the research project, and to ensure that in doing so we align with best practice wherever possible. The overarching areas of consideration are:

•     Ethical review of provenance and sourcing of the material

•     Legality of collection, transfer and use (national and international)

Each transfer of samples is further undertaken according to a Research Collaboration Agreement or Material Transfer Agreement entered into by the Darwin Tree of Life Partner, Genome Research Limited (operating as the Wellcome Sanger Institute), and in some circumstances other Darwin Tree of Life collaborators.

## Data Availability

European Nucleotide Archive: *Chenopodium album* (white goosefoot). Accession number PRJEB58230; https://identifiers.org/ena.embl/PRJEB58230 ( Wellcome Sanger Institute, 2023). The genome sequence is released openly for reuse. The *Chenopodium album* genome sequencing initiative is part of the Darwin Tree of Life (DToL) project. All raw sequence data and the assembly have been deposited in INSDC databases. The genome will be annotated using available RNA-Seq data and presented through the Ensembl pipeline at the European Bioinformatics Institute. Raw data and assembly accession identifiers are reported in Table 1.

## Author information

Members of the Royal Botanic Gardens Kew Genome Acquisition Lab are listed here: https://doi.org/10.5281/zenodo.12625079.

Members of the Plant Genome Sizing collective are listed here: https://doi.org/10.5281/zenodo.7994306.

Members of the Darwin Tree of Life Barcoding collective are listed here: https://doi.org/10.5281/zenodo.12158331

Members of the Wellcome Sanger Institute Tree of Life Management, Samples and Laboratory team are listed here: https://doi.org/10.5281/zenodo.12162482.

Members of Wellcome Sanger Institute Scientific Operations: Sequencing Operations are listed here: https://doi.org/10.5281/zenodo.12165051.

Members of the Wellcome Sanger Institute Tree of Life Core Informatics team are listed here: https://doi.org/10.5281/zenodo.12160324.

Members of the Tree of Life Core Informatics collective are listed here: https://doi.org/10.5281/zenodo.12205391.

Members of the Darwin Tree of Life Consortium are listed here: https://doi.org/10.5281/zenodo.4783558.
