# Supplementary material for: The genome sequence of fat-hen, Chenopodium album L
Source: Wellcome Open Res. 2024 Oct 15;9:508. Originally published 2024 Sep 4. [Version 2] doi: 10.12688/wellcomeopenres.23015.2 (PMC11452771; doi:10.12688/wellcomeopenres.23015.2)
Supplement: Peer review history

# Reviewer report: Reviewer response for version 2

**DOI:** 10.21956/wellcomeopenres.25599.r105067

**Published:** 2024-10-21

**Authors:** Do Hoang Dang Khoa

**Affiliations:** 1 Nguyen Tat Thanh University, Ho Chi Minh City, Vietnam

The authors revised the manuscript based on the reviewers' comments.

 I do not have more comments about the revised version.

 Thank you.

 a. Is the rationale for creating the dataset(s) clearly described?

 The processes for sampling, DNA extraction, sequencing, and analysis were described clearly. 

 b. Are the protocols appropriate and is the work technically sound?

 The protocol for obtaining the genomic data of Chenopodium album was appropriate, which included long reads, Hi-C, and RNA data. This study provided the genomic features of Chenopodium album which are necessary for further studies about the evolution of this species and its relatives.

 c. Are sufficient details of methods and materials provided to allow replication by others?

 The methods and materials were clearly described but it requires experienced scientists to replicate this study.

 d. Are the datasets clearly presented in a useable and accessible format?

 The datasets were available online so they could be reused.

Are sufficient details of methods and materials provided to allow replication by others?

Yes

Is the rationale for creating the dataset(s) clearly described?

Yes

Are the datasets clearly presented in a useable and accessible format?

Yes

Are the protocols appropriate and is the work technically sound?

Yes

Reviewer Expertise:

Plant Genomics, Sequencing methods, Plant Systematics.

I confirm that I have read this submission and believe that I have an appropriate level of expertise to confirm that it is of an acceptable scientific standard.

# Reviewer report: Reviewer response for version 2

**DOI:** 10.21956/wellcomeopenres.25599.r105068

**Published:** 2024-10-18

**Authors:** David E Jarvis

**Affiliations:** 1 Brigham Young University, Provo, USA

I do still wish that clarification were provided regarding the discrepancy between the assembly size (1.59 Gb) and the estimated genome size (2.04 Gb), and that sufficient details were provided in the Methods to enable independent replication, but the article is sufficient for a Data Note.

Are sufficient details of methods and materials provided to allow replication by others?

Partly

Is the rationale for creating the dataset(s) clearly described?

Yes

Are the datasets clearly presented in a useable and accessible format?

Partly

Are the protocols appropriate and is the work technically sound?

Yes

Reviewer Expertise:

Plant genetics and genomics

I confirm that I have read this submission and believe that I have an appropriate level of expertise to confirm that it is of an acceptable scientific standard.

# Reviewer report: Reviewer response for version 1

**DOI:** 10.21956/wellcomeopenres.25345.r101360

**Published:** 2024-10-04

**Authors:** Do Hoang Dang Khoa

**Affiliations:** 1 Nguyen Tat Thanh University, Ho Chi Minh City, Vietnam

The manuscript reported the genomic data of *Chenopodium album*.

 The content of the manuscript is suitable for DATA NOTE of Wellcome Open Research

 I have similar suggestions to the previous Reviewer, Dr. Rakesh Singh.

**Please check for other minor issues:**

 1. Please check the taxonomic status of *Chenopodium album* L.. According to Plant of the World Online, it belongs to Amaranthaceae (https://powo.science.kew.org/taxon/urn:lsid:ipni.org:names:55993-2).

 2. In the background section, please use italic font for *C. album*. Thank you.

**a. Is the rationale for creating the dataset(s) clearly described?**

 The processes for sampling, DNA extraction, sequencing, and analysis were described clearly. 

**b. Are the protocols appropriate and is the work technically sound?**

 The protocol for obtaining the genomic data of *Chenopodium album* was appropriate, which included long reads, Hi-C, and RNA data. This study provided the genomic features of *Chenopodium album* which are necessary for further studies about the evolution of this species and its relatives.

**c. Are sufficient details of methods and materials provided to allow replication by others?**

 The methods and materials were clearly described but it requires experienced scientists to replicate this study.

**d. Are the datasets clearly presented in a useable and accessible format?**

 The datasets were available online so they could be reused by other scientists.

Are sufficient details of methods and materials provided to allow replication by others?

Yes

Is the rationale for creating the dataset(s) clearly described?

Yes

Are the datasets clearly presented in a useable and accessible format?

Yes

Are the protocols appropriate and is the work technically sound?

Yes

Reviewer Expertise:

Plant Genomics, Sequencing methods, Plant Systematics.

I confirm that I have read this submission and believe that I have an appropriate level of expertise to confirm that it is of an acceptable scientific standard.

## References

[1] *Royal Botanic Gardens Kew;Plant of the World Online;Chenopodium album L.* . Reference source

# Reviewer report: Reviewer response for version 1

**DOI:** 10.21956/wellcomeopenres.25345.r99899

**Published:** 2024-10-04

**Authors:** David E Jarvis

**Affiliations:** 1 Brigham Young University, Provo, USA

This article reports the genome assembly of *Chenopodium album* L.

**Abstract**

 Should say "2n=6x=54" instead of "2n=2x=56."

**Genome sequence report**

 21.85 Gb of total sequencing data would represent about 14x coverage of a 1.59-Gb genome (or about 11x if using the 2.04 Gb estimate based on flow cytometry), not 53x as reported. Is the total sequencing data misreported, or is the fold coverage miscalculated? Table 1 reports three PacBio Sequel IIe runs, one of which produced 21.85 Gb of data; however, even adding the data from all three runs does not produce a fold coverage value of 53x.

 Data Note articles would obviously not be expected to have extensive descriptions of the data and results; however, additional explanation of the data in some cases is necessary to fully understand what was done and why. For example, results from a Blobtools analysis are presented in Figure 3, but the only mention of these results in the main text is “Figure 3 shows the base coverage against position in each chromosome.” Not only does this description incompletely report the full contents of Figure 3 (for example, in additional to read coverage, the figure also shows GC content and taxonomic affiliation for each sequence), it completely fails to address the reason for performing a Blobtools analysis and how the results may have been used to refine the genome assembly. Some small sequences show dramatically high read mapping coverage, and others have clearly higher GC contents. Were these sequences investigated to determine if they represent contaminant sequences? Many of these may likely represent portions of the chloroplast and/or mitochondrial genomes. If complete assemblies were produced for these genomes, were the contigs representing partial sequences removed from the final genome assembly?

 BUSCO suggests a largely complete assembly, and the k-mer completeness measure suggests that most of the sequence data is represented in the assembly; however, the assembly is only 78% of the estimated genome size based on flow cytometry. The authors should comment on this discrepancy. An additional estimate of genome size using k-mers would also be helpful. Completeness could also be assessed by comparing the assembly to genome assemblies from other related species, including tetraploid *Chenopodium quinoa* or hexaploid *Chenopodium formosanum*.

 The most informative way to name the chromosomes would be based on already-established chromosome names in the genus. Comparisons of the album chromosomes to related species and to themselves would enable identification of the sets of homologous chromosomes within the hexaploid genome.

**Methods**

 The Methods section is missing many details that would be necessary for someone else to independently replicate the results. For example, many of the programs used involve multiple steps and settings, but these details are not provided. For example, for genome annotation, were RNA-seq reads assembled into transcripts first? If so, how? Which “select set of proteins” from UniPort was used? Blobtools requires mapping reads to the reference and performing a homology search. How were these steps performed? Referring to the Assembly Screen for Cobionts and Contaminants (ASCC) pipeline, which hasn’t been published, does not enable someone else to replicate the results.

Are sufficient details of methods and materials provided to allow replication by others?

Partly

Is the rationale for creating the dataset(s) clearly described?

Yes

Are the datasets clearly presented in a useable and accessible format?

Partly

Are the protocols appropriate and is the work technically sound?

Yes

Reviewer Expertise:

Plant genetics and genomics

I confirm that I have read this submission and believe that I have an appropriate level of expertise to confirm that it is of an acceptable scientific standard, however I have significant reservations, as outlined above.

# Reviewer report: Reviewer response for version 1

**DOI:** 10.21956/wellcomeopenres.25345.r99128

**Published:** 2024-10-01

**Authors:** Rakesh Singh

**Affiliations:** 1 ICAR-National Bureau of Plant Genetic Resources, Delhi, India

Mian et al. presented a high-quality chromosome-level genome assembly of fat-hen, *Chenopodium album *L. Overall, the analysis is sufficient for the data note.

**Some minor improvements are suggested:**

- In the abstract, the authors mentioned the assembly is scaffolded into 27 chromosomal pseudomolecules, suggesting the individual is an allohexaploid (2 *n *= 6 *x *= 56). Please confirm allohexaploid distribution (2n = 6x = 54) or (2 *n *= 6 *x *= 56).
- In the background, the authors mentioned that *Chenopodium album *is also a problematic weed in agriculture, capable of causing major crop losses. It might be good to add a few more lines in this context and how genomics can be helpful to address this problem.
- A Comparison of the assembly structure with already published genomes of other closely related species, such as spinach ( *Spinacea oleracea*), will give an idea about how well the assembly has been performed.
- A phylogenetic tree can be drawn that shows the relationships between the *Chenopodium album* and other closely related species.
- The method section contains the names of software packages, their versions, references, and links to pipelines. It would be beneficial if it included all the detailed commands and parameters used, etc.

Are sufficient details of methods and materials provided to allow replication by others?

Yes

Is the rationale for creating the dataset(s) clearly described?

Yes

Are the datasets clearly presented in a useable and accessible format?

Yes

Are the protocols appropriate and is the work technically sound?

Yes

Reviewer Expertise:

Genomic Resources Generation in Horticultural Crops.

I confirm that I have read this submission and believe that I have an appropriate level of expertise to confirm that it is of an acceptable scientific standard.
